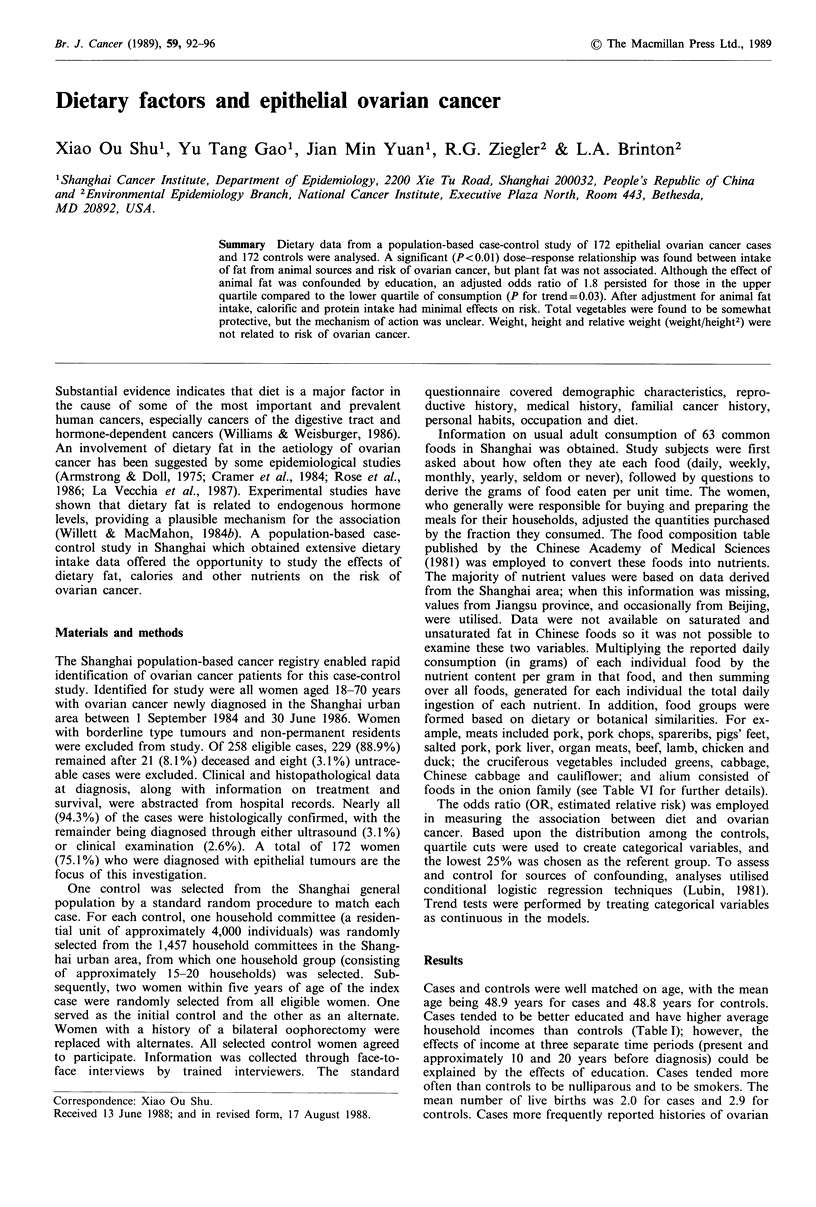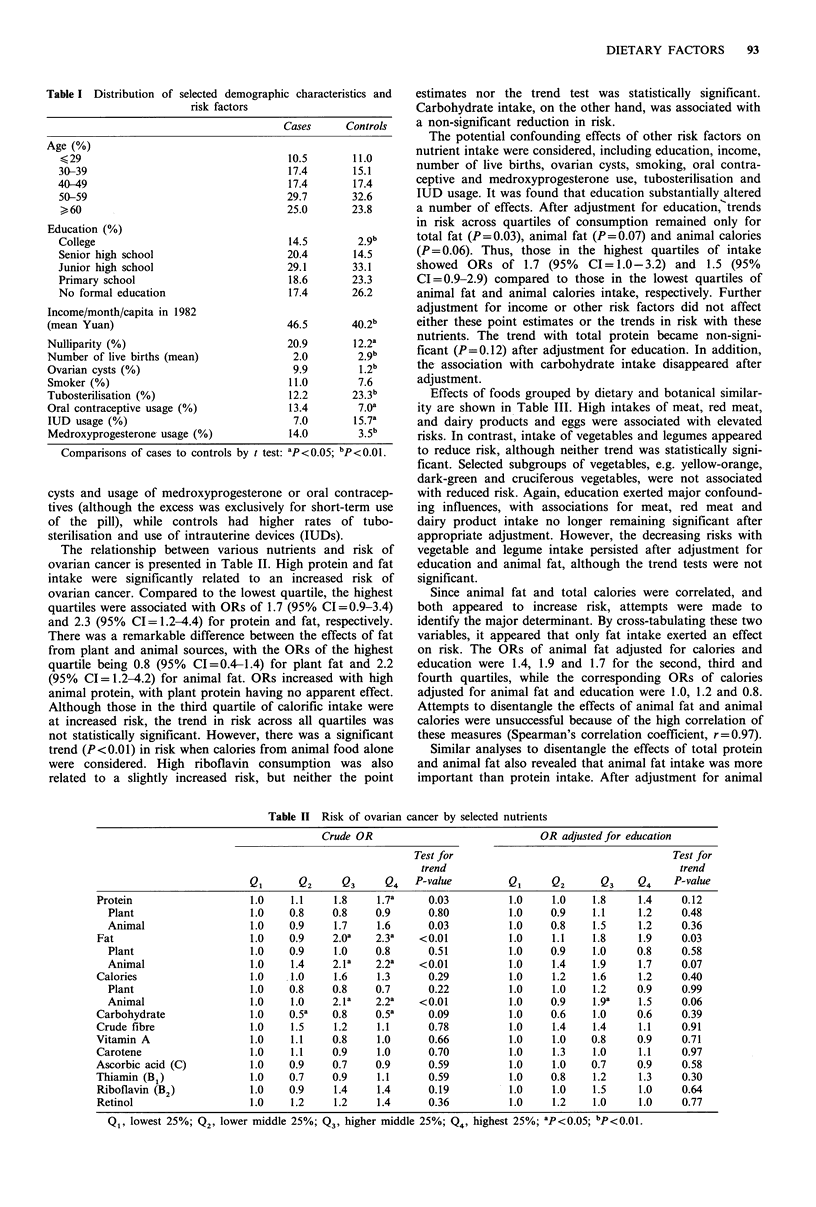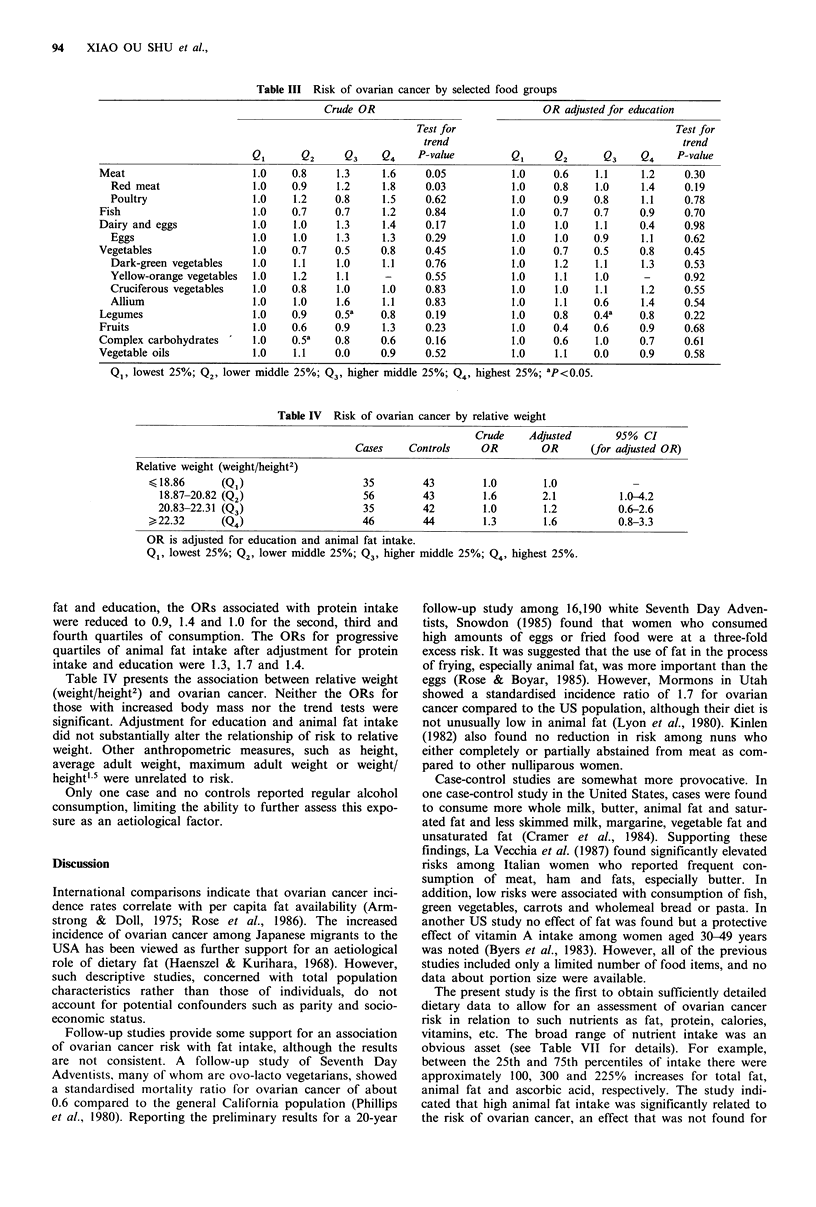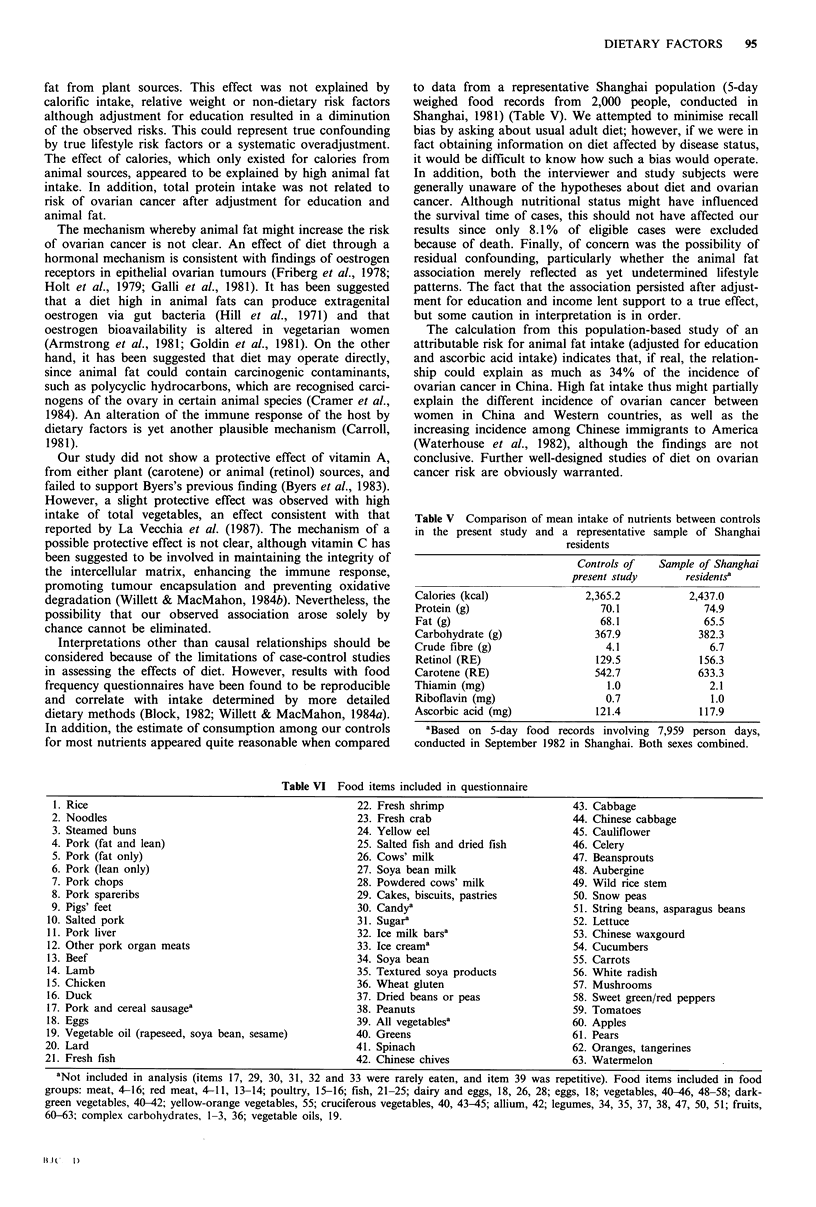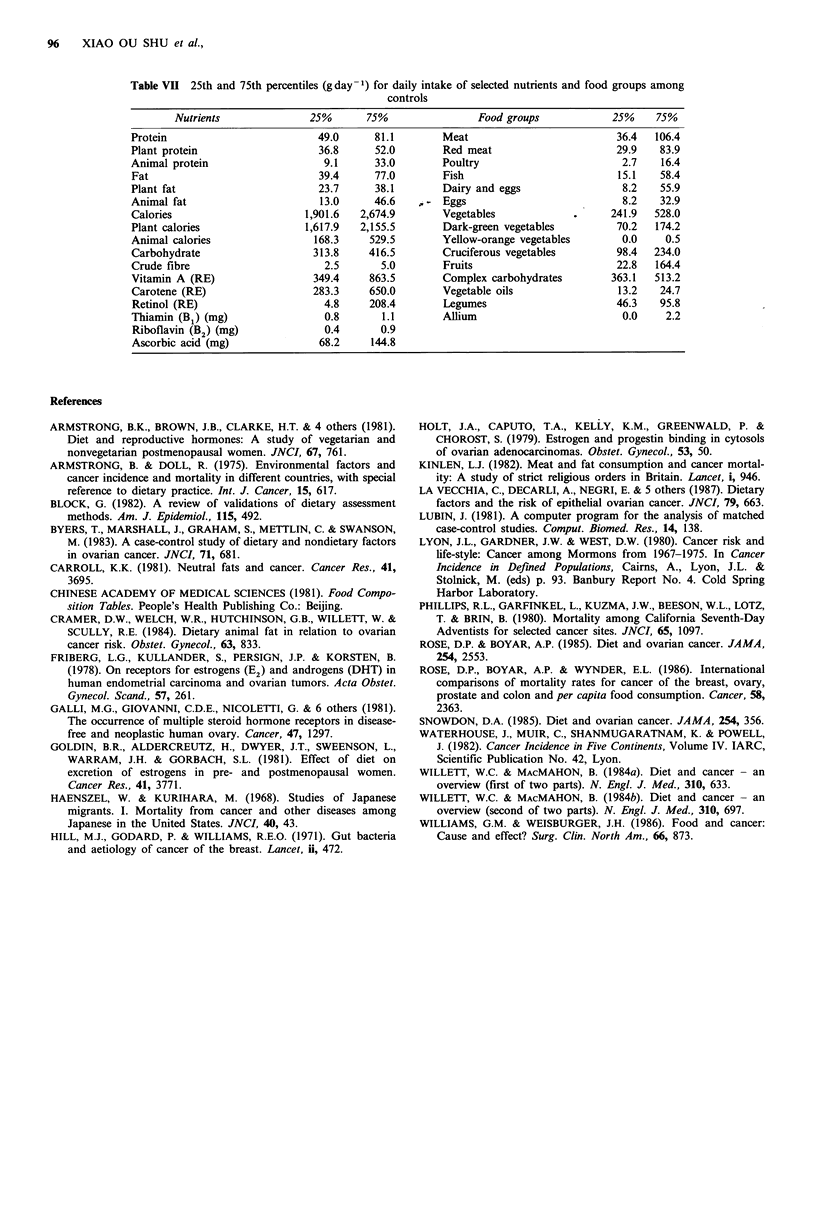# Dietary factors and epithelial ovarian cancer.

**DOI:** 10.1038/bjc.1989.18

**Published:** 1989-01

**Authors:** X. O. Shu, Y. T. Gao, J. M. Yuan, R. G. Ziegler, L. A. Brinton

**Affiliations:** Shanghai Cancer Institute, Department of Epidemiology, People's Republic of China.

## Abstract

Dietary data from a population-based case-control study of 172 epithelial ovarian cancer cases and 172 controls were analysed. A significant (P less than 0.01) dose-response relationship was found between intake of fat from animal sources and risk of ovarian cancer, but plant fat was not associated. Although the effect of animal fat was confounded by education, an adjusted odds ratio of 1.8 persisted for those in the upper quartile compared to the lower quartile of consumption (P for trend = 0.03). After adjustment for animal fat intake, calorific and protein intake had minimal effects on risk. Total vegetables were found to be somewhat protective, but the mechanism of action was unclear. Weight, height and relative weight (weight/height2) were not related to risk of ovarian cancer.


*Br. J. Cancer* (1989), **59**, 92–96 © The Macmillan Press Ltd., 1989

### Dietary factors and epithelial ovarian cancer

Xiao Ou Shu[1], Yu Tang Gao[1], Jian Min Yuan[1], R.G. Ziegler[2] & L.A. Brinton[2]

[1]*Shanghai Cancer Institute, Department of Epidemiology, 2200 Xie Tu Road, Shanghai 200032, People's Republic of China and* [2]*Environmental Epidemiology Branch, National Cancer Institute, Executive Plaza North, Room 443, Bethesda, MD 20892, USA.*

**Summary** Dietary data from a population-based case-control study of 172 epithelial ovarian cancer cases and 172 controls were analysed. A significant ($P<0.01$) dose–response relationship was found between intake of fat from animal sources and risk of ovarian cancer, but plant fat was not associated. Although the effect of animal fat was confounded by education, an adjusted odds ratio of 1.8 persisted for those in the upper quartile compared to the lower quartile of consumption ($P$ for trend$=0.03$). After adjustment for animal fat intake, calorific and protein intake had minimal effects on risk. Total vegetables were found to be somewhat protective, but the mechanism of action was unclear. Weight, height and relative weight (weight/height$^2$) were not related to risk of ovarian cancer.

Substantial evidence indicates that diet is a major factor in the cause of some of the most important and prevalent human cancers, especially cancers of the digestive tract and hormone-dependent cancers (Williams & Weisburger, 1986). An involvement of dietary fat in the aetiology of ovarian cancer has been suggested by some epidemiological studies (Armstrong & Doll, 1975; Cramer *et al.*, 1984; Rose *et al.*, 1986; La Vecchia *et al.*, 1987). Experimental studies have shown that dietary fat is related to endogenous hormone levels, providing a plausible mechanism for the association (Willett & MacMahon, 1984*b*). A population-based case-control study in Shanghai which obtained extensive dietary intake data offered the opportunity to study the effects of dietary fat, calories and other nutrients on the risk of ovarian cancer.

### Materials and methods

The Shanghai population-based cancer registry enabled rapid identification of ovarian cancer patients for this case-control study. Identified for study were all women aged 18–70 years with ovarian cancer newly diagnosed in the Shanghai urban area between 1 September 1984 and 30 June 1986. Women with borderline type tumours and non-permanent residents were excluded from study. Of 258 eligible cases, 229 (88.9%) remained after 21 (8.1%) deceased and eight (3.1%) untraceable cases were excluded. Clinical and histopathological data at diagnosis, along with information on treatment and survival, were abstracted from hospital records. Nearly all (94.3%) of the cases were histologically confirmed, with the remainder being diagnosed through either ultrasound (3.1%) or clinical examination (2.6%). A total of 172 women (75.1%) who were diagnosed with epithelial tumours are the focus of this investigation.

One control was selected from the Shanghai general population by a standard random procedure to match each case. For each control, one household committee (a residential unit of approximately 4,000 individuals) was randomly selected from the 1,457 household committees in the Shanghai urban area, from which one household group (consisting of approximately 15–20 households) was selected. Subsequently, two women within five years of age of the index case were randomly selected from all eligible women. One served as the initial control and the other as an alternate. Women with a history of a bilateral oophorectomy were replaced with alternates. All selected control women agreed to participate. Information was collected through face-to-face interviews by trained interviewers. The standard questionnaire covered demographic characteristics, reproductive history, medical history, familial cancer history, personal habits, occupation and diet.

Information on usual adult consumption of 63 common foods in Shanghai was obtained. Study subjects were first asked about how often they ate each food (daily, weekly, monthly, yearly, seldom or never), followed by questions to derive the grams of food eaten per unit time. The women, who generally were responsible for buying and preparing the meals for their households, adjusted the quantities purchased by the fraction they consumed. The food composition table published by the Chinese Academy of Medical Sciences (1981) was employed to convert these foods into nutrients. The majority of nutrient values were based on data derived from the Shanghai area; when this information was missing, values from Jiangsu province, and occasionally from Beijing, were utilised. Data were not available on saturated and unsaturated fat in Chinese foods so it was not possible to examine these two variables. Multiplying the reported daily consumption (in grams) of each individual food by the nutrient content per gram in that food, and then summing over all foods, generated for each individual the total daily ingestion of each nutrient. In addition, food groups were formed based on dietary or botanical similarities. For example, meats included pork, pork chops, spareribs, pigs' feet, salted pork, pork liver, organ meats, beef, lamb, chicken and duck; the cruciferous vegetables included greens, cabbage, Chinese cabbage and cauliflower; and alium consisted of foods in the onion family (see Table VI for further details).

The odds ratio (OR, estimated relative risk) was employed in measuring the association between diet and ovarian cancer. Based upon the distribution among the controls, quartile cuts were used to create categorical variables, and the lowest 25% was chosen as the referent group. To assess and control for sources of confounding, analyses utilised conditional logistic regression techniques (Lubin, 1981). Trend tests were performed by treating categorical variables as continuous in the models.

### Results

Cases and controls were well matched on age, with the mean age being 48.9 years for cases and 48.8 years for controls. Cases tended to be better educated and have higher average household incomes than controls (Table I); however, the effects of income at three separate time periods (present and approximately 10 and 20 years before diagnosis) could be explained by the effects of education. Cases tended more often than controls to be nulliparous and to be smokers. The mean number of live births was 2.0 for cases and 2.9 for controls. Cases more frequently reported histories of ovarian

Correspondence: Xiao Ou Shu.
Received 13 June 1988; and in revised form, 17 August 1988.

**Table I** Distribution of selected demographic characteristics and risk factors

| | *Cases* | *Controls* |
|---|---|---|
| Age (%) | | |
| ⩽29 | 10.5 | 11.0 |
| 30–39 | 17.4 | 15.1 |
| 40–49 | 17.4 | 17.4 |
| 50–59 | 29.7 | 32.6 |
| ⩾60 | 25.0 | 23.8 |
| Education (%) | | |
| College | 14.5 | 2.9[b] |
| Senior high school | 20.4 | 14.5 |
| Junior high school | 29.1 | 33.1 |
| Primary school | 18.6 | 23.3 |
| No formal education | 17.4 | 26.2 |
| Income/month/capita in 1982 (mean Yuan) | 46.5 | 40.2[b] |
| Nulliparity (%) | 20.9 | 12.2[a] |
| Number of live births (mean) | 2.0 | 2.9[b] |
| Ovarian cysts (%) | 9.9 | 1.2[b] |
| Smoker (%) | 11.0 | 7.6 |
| Tubosterilisation (%) | 12.2 | 23.3[b] |
| Oral contraceptive usage (%) | 13.4 | 7.0[a] |
| IUD usage (%) | 7.0 | 15.7[a] |
| Medroxyprogesterone usage (%) | 14.0 | 3.5[b] |

Comparisons of cases to controls by *t* test: [a]$P<0.05$; [b]$P<0.01$.

cysts and usage of medroxyprogesterone or oral contraceptives (although the excess was exclusively for short-term use of the pill), while controls had higher rates of tubosterilisation and use of intrauterine devices (IUDs).

The relationship between various nutrients and risk of ovarian cancer is presented in Table II. High protein and fat intake were significantly related to an increased risk of ovarian cancer. Compared to the lowest quartile, the highest quartiles were associated with ORs of 1.7 (95% CI = 0.9–3.4) and 2.3 (95% CI = 1.2–4.4) for protein and fat, respectively. There was a remarkable difference between the effects of fat from plant and animal sources, with the ORs of the highest quartile being 0.8 (95% CI = 0.4–1.4) for plant fat and 2.2 (95% CI = 1.2–4.2) for animal fat. ORs increased with high animal protein, with plant protein having no apparent effect. Although those in the third quartile of calorific intake were at increased risk, the trend in risk across all quartiles was not statistically significant. However, there was a significant trend ($P<0.01$) in risk when calories from animal food alone were considered. High riboflavin consumption was also related to a slightly increased risk, but neither the point estimates nor the trend test was statistically significant. Carbohydrate intake, on the other hand, was associated with a non-significant reduction in risk.

The potential confounding effects of other risk factors on nutrient intake were considered, including education, income, number of live births, ovarian cysts, smoking, oral contraceptive and medroxyprogesterone use, tubosterilisation and IUD usage. It was found that education substantially altered a number of effects. After adjustment for education, trends in risk across quartiles of consumption remained only for total fat ($P=0.03$), animal fat ($P=0.07$) and animal calories ($P=0.06$). Thus, those in the highest quartiles of intake showed ORs of 1.7 (95% CI = 1.0 − 3.2) and 1.5 (95% CI = 0.9–2.9) compared to those in the lowest quartiles of animal fat and animal calories intake, respectively. Further adjustment for income or other risk factors did not affect either these point estimates or the trends in risk with these nutrients. The trend with total protein became non-significant ($P=0.12$) after adjustment for education. In addition, the association with carbohydrate intake disappeared after adjustment.

Effects of foods grouped by dietary and botanical similarity are shown in Table III. High intakes of meat, red meat, and dairy products and eggs were associated with elevated risks. In contrast, intake of vegetables and legumes appeared to reduce risk, although neither trend was statistically significant. Selected subgroups of vegetables, e.g. yellow-orange, dark-green and cruciferous vegetables, were not associated with reduced risk. Again, education exerted major confounding influences, with associations for meat, red meat and dairy product intake no longer remaining significant after appropriate adjustment. However, the decreasing risks with vegetable and legume intake persisted after adjustment for education and animal fat, although the trend tests were not significant.

Since animal fat and total calories were correlated, and both appeared to increase risk, attempts were made to identify the major determinant. By cross-tabulating these two variables, it appeared that only fat intake exerted an effect on risk. The ORs of animal fat adjusted for calories and education were 1.4, 1.9 and 1.7 for the second, third and fourth quartiles, while the corresponding ORs of calories adjusted for animal fat and education were 1.0, 1.2 and 0.8. Attempts to disentangle the effects of animal fat and animal calories were unsuccessful because of the high correlation of these measures (Spearman's correlation coefficient, $r=0.97$).

Similar analyses to disentangle the effects of total protein and animal fat also revealed that animal fat intake was more important than protein intake. After adjustment for animal

**Table II** Risk of ovarian cancer by selected nutrients

| | *Crude OR* | | | | | *OR adjusted for education* | | | | |
|---|---|---|---|---|---|---|---|---|---|---|
| | $Q_1$ | $Q_2$ | $Q_3$ | $Q_4$ | *Test for trend P-value* | $Q_1$ | $Q_2$ | $Q_3$ | $Q_4$ | *Test for trend P-value* |
| Protein | 1.0 | 1.1 | 1.8 | 1.7[a] | 0.03 | 1.0 | 1.0 | 1.8 | 1.4 | 0.12 |
| Plant | 1.0 | 0.8 | 0.8 | 0.9 | 0.80 | 1.0 | 0.9 | 1.1 | 1.2 | 0.48 |
| Animal | 1.0 | 0.9 | 1.7 | 1.6 | 0.03 | 1.0 | 0.8 | 1.5 | 1.2 | 0.36 |
| Fat | 1.0 | 0.9 | 2.0[a] | 2.3[a] | <0.01 | 1.0 | 1.1 | 1.8 | 1.9 | 0.03 |
| Plant | 1.0 | 0.9 | 1.0 | 0.8 | 0.51 | 1.0 | 0.9 | 1.0 | 0.8 | 0.58 |
| Animal | 1.0 | 1.4 | 2.1[a] | 2.2[a] | <0.01 | 1.0 | 1.4 | 1.9 | 1.7 | 0.07 |
| Calories | 1.0 | 1.0 | 1.6 | 1.3 | 0.29 | 1.0 | 1.2 | 1.6 | 1.2 | 0.40 |
| Plant | 1.0 | 0.8 | 0.8 | 0.7 | 0.22 | 1.0 | 1.0 | 1.2 | 0.9 | 0.99 |
| Animal | 1.0 | 1.0 | 2.1[a] | 2.2[a] | <0.01 | 1.0 | 0.9 | 1.9[a] | 1.5 | 0.06 |
| Carbohydrate | 1.0 | 0.5[a] | 0.8 | 0.5[a] | 0.09 | 1.0 | 0.6 | 1.0 | 0.6 | 0.39 |
| Crude fibre | 1.0 | 1.5 | 1.2 | 1.1 | 0.78 | 1.0 | 1.4 | 1.4 | 1.1 | 0.91 |
| Vitamin A | 1.0 | 1.1 | 0.8 | 1.0 | 0.66 | 1.0 | 1.0 | 0.8 | 0.9 | 0.71 |
| Carotene | 1.0 | 1.1 | 0.9 | 1.0 | 0.70 | 1.0 | 1.3 | 1.0 | 1.1 | 0.97 |
| Ascorbic acid (C) | 1.0 | 0.9 | 0.7 | 0.9 | 0.59 | 1.0 | 1.0 | 0.7 | 0.9 | 0.58 |
| Thiamin ($B_1$) | 1.0 | 0.7 | 0.9 | 1.1 | 0.59 | 1.0 | 0.8 | 1.2 | 1.3 | 0.30 |
| Riboflavin ($B_2$) | 1.0 | 0.9 | 1.4 | 1.4 | 0.19 | 1.0 | 1.0 | 1.5 | 1.0 | 0.64 |
| Retinol | 1.0 | 1.2 | 1.2 | 1.4 | 0.36 | 1.0 | 1.2 | 1.0 | 1.0 | 0.77 |

$Q_1$, lowest 25%; $Q_2$, lower middle 25%; $Q_3$, higher middle 25%; $Q_4$, highest 25%; [a]$P<0.05$; [b]$P<0.01$.

**Table III** Risk of ovarian cancer by selected food groups

| | *Crude OR* | | | | | *OR adjusted for education* | | | | |
|---|---|---|---|---|---|---|---|---|---|---|
| | $Q_1$ | $Q_2$ | $Q_3$ | $Q_4$ | *Test for trend P-value* | $Q_1$ | $Q_2$ | $Q_3$ | $Q_4$ | *Test for trend P-value* |
| Meat | 1.0 | 0.8 | 1.3 | 1.6 | 0.05 | 1.0 | 0.6 | 1.1 | 1.2 | 0.30 |
| Red meat | 1.0 | 0.9 | 1.2 | 1.8 | 0.03 | 1.0 | 0.8 | 1.0 | 1.4 | 0.19 |
| Poultry | 1.0 | 1.2 | 0.8 | 1.5 | 0.62 | 1.0 | 0.9 | 0.8 | 1.1 | 0.78 |
| Fish | 1.0 | 0.7 | 0.7 | 1.2 | 0.84 | 1.0 | 0.7 | 0.7 | 0.9 | 0.70 |
| Dairy and eggs | 1.0 | 1.0 | 1.3 | 1.4 | 0.17 | 1.0 | 1.0 | 1.1 | 0.4 | 0.98 |
| Eggs | 1.0 | 1.0 | 1.3 | 1.3 | 0.29 | 1.0 | 1.0 | 0.9 | 1.1 | 0.62 |
| Vegetables | 1.0 | 0.7 | 0.5 | 0.8 | 0.45 | 1.0 | 0.7 | 0.5 | 0.8 | 0.45 |
| Dark-green vegetables | 1.0 | 1.1 | 1.0 | 1.1 | 0.76 | 1.0 | 1.2 | 1.1 | 1.3 | 0.53 |
| Yellow-orange vegetables | 1.0 | 1.2 | 1.1 | – | 0.55 | 1.0 | 1.1 | 1.0 | – | 0.92 |
| Cruciferous vegetables | 1.0 | 0.8 | 1.0 | 1.0 | 0.83 | 1.0 | 1.0 | 1.1 | 1.2 | 0.55 |
| Allium | 1.0 | 1.0 | 1.6 | 1.1 | 0.83 | 1.0 | 1.1 | 0.6 | 1.4 | 0.54 |
| Legumes | 1.0 | 0.9 | 0.5[a] | 0.8 | 0.19 | 1.0 | 0.8 | 0.4[a] | 0.8 | 0.22 |
| Fruits | 1.0 | 0.6 | 0.9 | 1.3 | 0.23 | 1.0 | 0.4 | 0.6 | 0.9 | 0.68 |
| Complex carbohydrates | 1.0 | 0.5[a] | 0.8 | 0.6 | 0.16 | 1.0 | 0.6 | 1.0 | 0.7 | 0.61 |
| Vegetable oils | 1.0 | 1.1 | 0.0 | 0.9 | 0.52 | 1.0 | 1.1 | 0.0 | 0.9 | 0.58 |

$Q_1$, lowest 25%; $Q_2$, lower middle 25%; $Q_3$, higher middle 25%; $Q_4$, highest 25%; [a]$P<0.05$.

**Table IV** Risk of ovarian cancer by relative weight

| | *Cases* | *Controls* | *Crude OR* | *Adjusted OR* | *95% CI (for adjusted OR)* |
|---|---|---|---|---|---|
| Relative weight (weight/height$^2$) | | | | | |
| ≤18.86 ($Q_1$) | 35 | 43 | 1.0 | 1.0 | – |
| 18.87–20.82 ($Q_2$) | 56 | 43 | 1.6 | 2.1 | 1.0–4.2 |
| 20.83–22.31 ($Q_3$) | 35 | 42 | 1.0 | 1.2 | 0.6–2.6 |
| ≥22.32 ($Q_4$) | 46 | 44 | 1.3 | 1.6 | 0.8–3.3 |

OR is adjusted for education and animal fat intake.
$Q_1$, lowest 25%; $Q_2$, lower middle 25%; $Q_3$, higher middle 25%; $Q_4$, highest 25%.

fat and education, the ORs associated with protein intake were reduced to 0.9, 1.4 and 1.0 for the second, third and fourth quartiles of consumption. The ORs for progressive quartiles of animal fat intake after adjustment for protein intake and education were 1.3, 1.7 and 1.4.

Table IV presents the association between relative weight (weight/height$^2$) and ovarian cancer. Neither the ORs for those with increased body mass nor the trend tests were significant. Adjustment for education and animal fat intake did not substantially alter the relationship of risk to relative weight. Other anthropometric measures, such as height, average adult weight, maximum adult weight or weight/height$^{1.5}$ were unrelated to risk.

Only one case and no controls reported regular alcohol consumption, limiting the ability to further assess this exposure as an aetiological factor.

### Discussion

International comparisons indicate that ovarian cancer incidence rates correlate with per capita fat availability (Armstrong & Doll, 1975; Rose *et al.*, 1986). The increased incidence of ovarian cancer among Japanese migrants to the USA has been viewed as further support for an aetiological role of dietary fat (Haenszel & Kurihara, 1968). However, such descriptive studies, concerned with total population characteristics rather than those of individuals, do not account for potential confounders such as parity and socio-economic status.

Follow-up studies provide some support for an association of ovarian cancer risk with fat intake, although the results are not consistent. A follow-up study of Seventh Day Adventists, many of whom are ovo-lacto vegetarians, showed a standardised mortality ratio for ovarian cancer of about 0.6 compared to the general California population (Phillips *et al.*, 1980). Reporting the preliminary results for a 20-year follow-up study among 16,190 white Seventh Day Adventists, Snowdon (1985) found that women who consumed high amounts of eggs or fried food were at a three-fold excess risk. It was suggested that the use of fat in the process of frying, especially animal fat, was more important than the eggs (Rose & Boyar, 1985). However, Mormons in Utah showed a standardised incidence ratio of 1.7 for ovarian cancer compared to the US population, although their diet is not unusually low in animal fat (Lyon *et al.*, 1980). Kinlen (1982) also found no reduction in risk among nuns who either completely or partially abstained from meat as compared to other nulliparous women.

Case-control studies are somewhat more provocative. In one case-control study in the United States, cases were found to consume more whole milk, butter, animal fat and saturated fat and less skimmed milk, margarine, vegetable fat and unsaturated fat (Cramer *et al.*, 1984). Supporting these findings, La Vecchia *et al.* (1987) found significantly elevated risks among Italian women who reported frequent consumption of meat, ham and fats, especially butter. In addition, low risks were associated with consumption of fish, green vegetables, carrots and wholemeal bread or pasta. In another US study no effect of fat was found but a protective effect of vitamin A intake among women aged 30–49 years was noted (Byers *et al.*, 1983). However, all of the previous studies included only a limited number of food items, and no data about portion size were available.

The present study is the first to obtain sufficiently detailed dietary data to allow for an assessment of ovarian cancer risk in relation to such nutrients as fat, protein, calories, vitamins, etc. The broad range of nutrient intake was an obvious asset (see Table VII for details). For example, between the 25th and 75th percentiles of intake there were approximately 100, 300 and 225% increases for total fat, animal fat and ascorbic acid, respectively. The study indicated that high animal fat intake was significantly related to the risk of ovarian cancer, an effect that was not found for fat from plant sources. This effect was not explained by calorific intake, relative weight or non-dietary risk factors although adjustment for education resulted in a diminution of the observed risks. This could represent true confounding by true lifestyle risk factors or a systematic overadjustment. The effect of calories, which only existed for calories from animal sources, appeared to be explained by high animal fat intake. In addition, total protein intake was not related to risk of ovarian cancer after adjustment for education and animal fat.

The mechanism whereby animal fat might increase the risk of ovarian cancer is not clear. An effect of diet through a hormonal mechanism is consistent with findings of oestrogen receptors in epithelial ovarian tumours (Friberg *et al.*, 1978; Holt *et al.*, 1979; Galli *et al.*, 1981). It has been suggested that a diet high in animal fats can produce extragenital oestrogen via gut bacteria (Hill *et al.*, 1971) and that oestrogen bioavailability is altered in vegetarian women (Armstrong *et al.*, 1981; Goldin *et al.*, 1981). On the other hand, it has been suggested that diet may operate directly, since animal fat could contain carcinogenic contaminants, such as polycyclic hydrocarbons, which are recognised carcinogens of the ovary in certain animal species (Cramer *et al.*, 1984). An alteration of the immune response of the host by dietary factors is yet another plausible mechanism (Carroll, 1981).

Our study did not show a protective effect of vitamin A, from either plant (carotene) or animal (retinol) sources, and failed to support Byers's previous finding (Byers *et al.*, 1983). However, a slight protective effect was observed with high intake of total vegetables, an effect consistent with that reported by La Vecchia *et al.* (1987). The mechanism of a possible protective effect is not clear, although vitamin C has been suggested to be involved in maintaining the integrity of the intercellular matrix, enhancing the immune response, promoting tumour encapsulation and preventing oxidative degradation (Willett & MacMahon, 1984*b*). Nevertheless, the possibility that our observed association arose solely by chance cannot be eliminated.

Interpretations other than causal relationships should be considered because of the limitations of case-control studies in assessing the effects of diet. However, results with food frequency questionnaires have been found to be reproducible and correlate with intake determined by more detailed dietary methods (Block, 1982; Willett & MacMahon, 1984*a*). In addition, the estimate of consumption among our controls for most nutrients appeared quite reasonable when compared to data from a representative Shanghai population (5-day weighed food records from 2,000 people, conducted in Shanghai, 1981) (Table V). We attempted to minimise recall bias by asking about usual adult diet; however, if we were in fact obtaining information on diet affected by disease status, it would be difficult to know how such a bias would operate. In addition, both the interviewer and study subjects were generally unaware of the hypotheses about diet and ovarian cancer. Although nutritional status might have influenced the survival time of cases, this should not have affected our results since only 8.1% of eligible cases were excluded because of death. Finally, of concern was the possibility of residual confounding, particularly whether the animal fat association merely reflected as yet undetermined lifestyle patterns. The fact that the association persisted after adjustment for education and income lent support to a true effect, but some caution in interpretation is in order.

The calculation from this population-based study of an attributable risk for animal fat intake (adjusted for education and ascorbic acid intake) indicates that, if real, the relationship could explain as much as 34% of the incidence of ovarian cancer in China. High fat intake thus might partially explain the different incidence of ovarian cancer between women in China and Western countries, as well as the increasing incidence among Chinese immigrants to America (Waterhouse *et al.*, 1982), although the findings are not conclusive. Further well-designed studies of diet on ovarian cancer risk are obviously warranted.

**Table V** Comparison of mean intake of nutrients between controls in the present study and a representative sample of Shanghai residents

| | *Controls of present study* | *Sample of Shanghai residents*[a] |
|---|---|---|
| Calories (kcal) | 2,365.2 | 2,437.0 |
| Protein (g) | 70.1 | 74.9 |
| Fat (g) | 68.1 | 65.5 |
| Carbohydrate (g) | 367.9 | 382.3 |
| Crude fibre (g) | 4.1 | 6.7 |
| Retinol (RE) | 129.5 | 156.3 |
| Carotene (RE) | 542.7 | 633.3 |
| Thiamin (mg) | 1.0 | 2.1 |
| Riboflavin (mg) | 0.7 | 1.0 |
| Ascorbic acid (mg) | 121.4 | 117.9 |

[a]Based on 5-day food records involving 7,959 person days, conducted in September 1982 in Shanghai. Both sexes combined.

**Table VI** Food items included in questionnaire

| | | |
|---|---|---|
| 1. Rice | 22. Fresh shrimp | 43. Cabbage |
| 2. Noodles | 23. Fresh crab | 44. Chinese cabbage |
| 3. Steamed buns | 24. Yellow eel | 45. Cauliflower |
| 4. Pork (fat and lean) | 25. Salted fish and dried fish | 46. Celery |
| 5. Pork (fat only) | 26. Cows' milk | 47. Beansprouts |
| 6. Pork (lean only) | 27. Soya bean milk | 48. Aubergine |
| 7. Pork chops | 28. Powdered cows' milk | 49. Wild rice stem |
| 8. Pork spareribs | 29. Cakes, biscuits, pastries | 50. Snow peas |
| 9. Pigs' feet | 30. Candy[a] | 51. String beans, asparagus beans |
| 10. Salted pork | 31. Sugar[a] | 52. Lettuce |
| 11. Pork liver | 32. Ice milk bars[a] | 53. Chinese waxgourd |
| 12. Other pork organ meats | 33. Ice cream[a] | 54. Cucumbers |
| 13. Beef | 34. Soya bean | 55. Carrots |
| 14. Lamb | 35. Textured soya products | 56. White radish |
| 15. Chicken | 36. Wheat gluten | 57. Mushrooms |
| 16. Duck | 37. Dried beans or peas | 58. Sweet green/red peppers |
| 17. Pork and cereal sausage[a] | 38. Peanuts | 59. Tomatoes |
| 18. Eggs | 39. All vegetables[a] | 60. Apples |
| 19. Vegetable oil (rapeseed, soya bean, sesame) | 40. Greens | 61. Pears |
| 20. Lard | 41. Spinach | 62. Oranges, tangerines |
| 21. Fresh fish | 42. Chinese chives | 63. Watermelon |

[a]Not included in analysis (items 17, 29, 30, 31, 32 and 33 were rarely eaten, and item 39 was repetitive). Food items included in food groups: meat, 4–16; red meat, 4–11, 13–14; poultry, 15–16; fish, 21–25; dairy and eggs, 18, 26, 28; eggs, 18; vegetables, 40–46, 48–58; dark-green vegetables, 40–42; yellow-orange vegetables, 55; cruciferous vegetables, 40, 43–45; allium, 42; legumes, 34, 35, 37, 38, 47, 50, 51; fruits, 60–63; complex carbohydrates, 1–3, 36; vegetable oils, 19.

**Table VII** 25th and 75th percentiles (g day$^{-1}$) for daily intake of selected nutrients and food groups among controls

| *Nutrients* | *25%* | *75%* | *Food groups* | *25%* | *75%* |
|---|---|---|---|---|---|
| Protein | 49.0 | 81.1 | Meat | 36.4 | 106.4 |
| Plant protein | 36.8 | 52.0 | Red meat | 29.9 | 83.9 |
| Animal protein | 9.1 | 33.0 | Poultry | 2.7 | 16.4 |
| Fat | 39.4 | 77.0 | Fish | 15.1 | 58.4 |
| Plant fat | 23.7 | 38.1 | Dairy and eggs | 8.2 | 55.9 |
| Animal fat | 13.0 | 46.6 | Eggs | 8.2 | 32.9 |
| Calories | 1,901.6 | 2,674.9 | Vegetables | 241.9 | 528.0 |
| Plant calories | 1,617.9 | 2,155.5 | Dark-green vegetables | 70.2 | 174.2 |
| Animal calories | 168.3 | 529.5 | Yellow-orange vegetables | 0.0 | 0.5 |
| Carbohydrate | 313.8 | 416.5 | Cruciferous vegetables | 98.4 | 234.0 |
| Crude fibre | 2.5 | 5.0 | Fruits | 22.8 | 164.4 |
| Vitamin A (RE) | 349.4 | 863.5 | Complex carbohydrates | 363.1 | 513.2 |
| Carotene (RE) | 283.3 | 650.0 | Vegetable oils | 13.2 | 24.7 |
| Retinol (RE) | 4.8 | 208.4 | Legumes | 46.3 | 95.8 |
| Thiamin ($B_1$) (mg) | 0.8 | 1.1 | Allium | 0.0 | 2.2 |
| Riboflavin ($B_2$) (mg) | 0.4 | 0.9 | | | |
| Ascorbic acid (mg) | 68.2 | 144.8 | | | |

### References

ARMSTRONG, B.K., BROWN, J.B., CLARKE, H.T. & 4 others (1981). Diet and reproductive hormones: A study of vegetarian and nonvegetarian postmenopausal women. *JNCI*, **67**, 761.

ARMSTRONG, B. & DOLL, R. (1975). Environmental factors and cancer incidence and mortality in different countries, with special reference to dietary practice. *Int. J. Cancer*, **15**, 617.

BLOCK, G. (1982). A review of validations of dietary assessment methods. *Am. J. Epidemiol.*, **115**, 492.

BYERS, T., MARSHALL, J., GRAHAM, S., METTLIN, C. & SWANSON, M. (1983). A case-control study of dietary and nondietary factors in ovarian cancer. *JNCI*, **71**, 681.

CARROLL, K.K. (1981). Neutral fats and cancer. *Cancer Res.*, **41**, 3695.

CHINESE ACADEMY OF MEDICAL SCIENCES (1981). *Food Composition Tables*. People's Health Publishing Co.: Beijing.

CRAMER, D.W., WELCH, W.R., HUTCHINSON, G.B., WILLETT, W. & SCULLY, R.E. (1984). Dietary animal fat in relation to ovarian cancer risk. *Obstet. Gynecol.*, **63**, 833.

FRIBERG, L.G., KULLANDER, S., PERSIGN, J.P. & KORSTEN, B. (1978). On receptors for estrogens ($E_2$) and androgens (DHT) in human endometrial carcinoma and ovarian tumors. *Acta Obstet. Gynecol. Scand.*, **57**, 261.

GALLI, M.G., GIOVANNI, C.D.E., NICOLETTI, G. & 6 others (1981). The occurrence of multiple steroid hormone receptors in disease-free and neoplastic human ovary. *Cancer*, **47**, 1297.

GOLDIN, B.R., ALDERCREUTZ, H., DWYER, J.T., SWEENSON, L., WARRAM, J.H. & GORBACH, S.L. (1981). Effect of diet on excretion of estrogens in pre- and postmenopausal women. *Cancer Res.*, **41**, 3771.

HAENSZEL, W. & KURIHARA, M. (1968). Studies of Japanese migrants. I. Mortality from cancer and other diseases among Japanese in the United States. *JNCI*, **40**, 43.

HILL, M.J., GODARD, P. & WILLIAMS, R.E.O. (1971). Gut bacteria and aetiology of cancer of the breast. *Lancet*, **ii**, 472.

HOLT, J.A., CAPUTO, T.A., KELLY, K.M., GREENWALD, P. & CHOROST, S. (1979). Estrogen and progestin binding in cytosols of ovarian adenocarcinomas. *Obstet. Gynecol.*, **53**, 50.

KINLEN, L.J. (1982). Meat and fat consumption and cancer mortality: A study of strict religious orders in Britain. *Lancet*, **i**, 946.

LA VECCHIA, C., DECARLI, A., NEGRI, E. & 5 others (1987). Dietary factors and the risk of epithelial ovarian cancer. *JNCI*, **79**, 663.

LUBIN, J. (1981). A computer program for the analysis of matched case-control studies. *Comput. Biomed. Res.*, **14**, 138.

LYON, J.L., GARDNER, J.W. & WEST, D.W. (1980). Cancer risk and life-style: Cancer among Mormons from 1967–1975. In *Cancer Incidence in Defined Populations*, Cairns, A., Lyon, J.L. & Stolnick, M. (eds) p. 93. Banbury Report No. 4. Cold Spring Harbor Laboratory.

PHILLIPS, R.L., GARFINKEL, L., KUZMA, J.W., BEESON, W.L., LOTZ, T. & BRIN, B. (1980). Mortality among California Seventh-Day Adventists for selected cancer sites. *JNCI*, **65**, 1097.

ROSE, D.P. & BOYAR, A.P. (1985). Diet and ovarian cancer. *JAMA*, **254**, 2553.

ROSE, D.P., BOYAR, A.P. & WYNDER, E.L. (1986). International comparisons of mortality rates for cancer of the breast, ovary, prostate and colon and *per capita* food consumption. *Cancer*, **58**, 2363.

SNOWDON, D.A. (1985). Diet and ovarian cancer. *JAMA*, **254**, 356.

WATERHOUSE, J., MUIR, C., SHANMUGARATNAM, K. & POWELL, J. (1982). *Cancer Incidence in Five Continents*, Volume IV. IARC, Scientific Publication No. 42, Lyon.

WILLETT, W.C. & MACMAHON, B. (1984*a*). Diet and cancer – an overview (first of two parts). *N. Engl. J. Med.*, **310**, 633.

WILLETT, W.C. & MACMAHON, B. (1984*b*). Diet and cancer – an overview (second of two parts). *N. Engl. J. Med.*, **310**, 697.

WILLIAMS, G.M. & WEISBURGER, J.H. (1986). Food and cancer: Cause and effect? *Surg. Clin. North Am.*, **66**, 873.